# Endocardial and Epicardial Rhythmia HDx™ Mapping Verifies Surgical Cox Maze IV Lesion Pattern

**DOI:** 10.19102/icrm.2020.110104

**Published:** 2020-01-15

**Authors:** Alexandra L. Kharazi, Frank Villa Hernandez, J. Paul Mounsey, Andy C. Kiser

**Affiliations:** ^1^Department of Cardiovascular Sciences, East Carolina University, Greenville, NC, USA; ^2^Cardiovascular Services, St. Clair Hospital, Pittsburgh, PA, USA

**Keywords:** Arrythmias, atrial fibrillation, cardiac, cardiac surgery, electrophysiology, inferior vena cava

## Abstract

Atrial fibrillation (AF) remains the most common cardiac dysarrhythmia, with a significant impact on the health-care economy. AF occurs as a result of electrical conduction delays within the atrial tissue, which may stem from fibrosis or other mediators that alter atrial tissue conduction substrate. The Cox maze III and IV procedures block these reentry circuits by directly dividing, or breaking, the self-perpetuating circuit and by isolating these circuits away from the intrinsic cardiac conduction pathway. The Convergent procedure, a hybrid approach to AF ablation, coordinates the electrophysiologist and the cardiac surgeon in a simultaneous or sequential endocardial and epicardial procedure. Because the heart remains electrically active, electroanatomical maps, not anatomical landmarks, guide therapy. However, lesion transmurality and contiguity remain inconsistent. The Rhythmia HDx™ mapping system (Boston Scientific, Natick, MA, USA) offers detailed maps of acute lesion patterns during the ablation procedure. However, these maps require instrumentation and technology found in the electrophysiology laboratory, not in the operating room. We herein present a case during which we performed a Cox maze IV procedure and also applied the Rhythmia HDx™ electroanatomical mapping system (Boston Scientific, Natick, MA, USA) at the time of operation. Through this novel approach, we were able to verify the completeness of the lesions created and reach a procedural endpoint confirmed by both epicardial and endocardial maps of successful electrical isolation of the left atrium posterior wall and pulmonary vein pedicle.

## Introduction

Atrial fibrillation (AF) remains the most common cardiac arrhythmia, placing a significant burden on the health-care economy. AF occurs as a result of electrical conduction delays within the atrial tissue, which may stem from fibrosis or other mediators that alter atrial tissue conduction substrate. The Cox maze III and IV^1,2^ procedures block these reentry circuits by directly dividing (or breaking) the self-perpetuating circuit and by isolating these circuits away from the intrinsic cardiac conduction pathway. The Convergent procedure,^3^ a hybrid approach to AF ablation, coordinates the actions of the electrophysiologist and the cardiac surgeon together in a simultaneous or sequential endocardial and epicardial procedure. Because the heart remains electrically active, electroanatomical maps, not anatomical landmarks, guide therapy. However, lesion transmurality and contiguity remain inconsistent.^4^ The Rhythmia HDx™ mapping system (Boston Scientific, Natick, MA, USA) provides detailed maps of the acute lesion pattern during the ablation. These maps require instrumentation and technology found in the electrophysiology laboratory, not in the operating room. This case discussion demonstrates physician collaboration and technology integration by deploying the Rhythmia™ HDx™ mapping system (Boston Scientific, Natick, MA, USA) in the operating room to document acute lesion contiguity and transmurality of the Cox maze IV procedure.

## Case presentation

**Figure 1:**
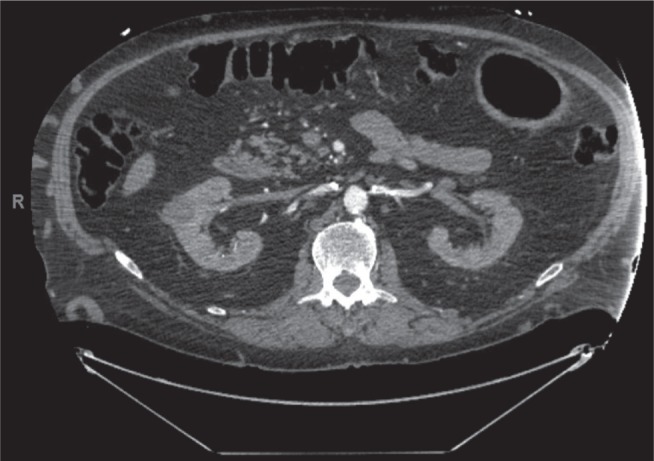
Abdominal CT with contrast showing the abdominal aorta with renal artery branches. Renal veins are also seen, but the IVC is absent.

**Figure 2:**
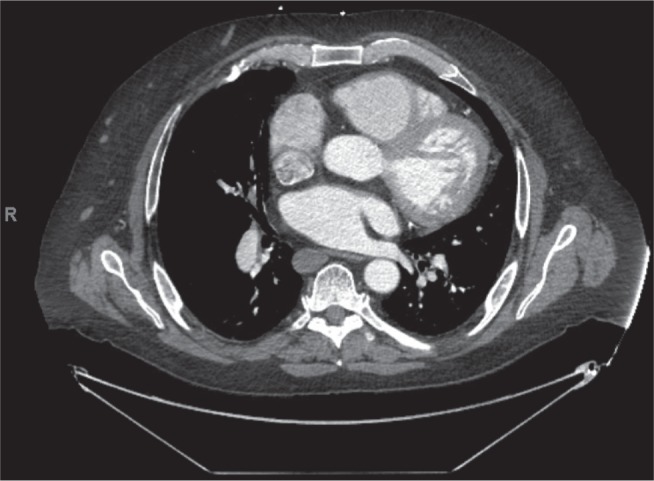
Chest CT with contrast demonstrating the SVC feeding into the right atrium. The descending aorta is seen, but the IVC is not visualized.

**Figure 3:**
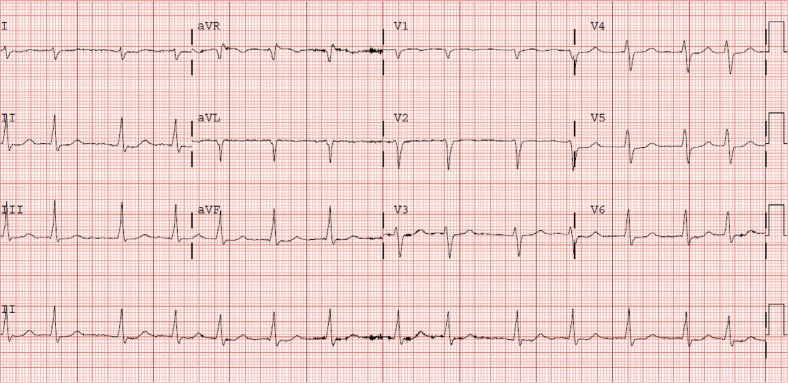
Electrocardiogram displaying an absence of P-waves and irregular R–R intervals indicating the presence of AF.

A 60-year-old male with a congenital absence of the inferior vena cava (IVC) **(Figures 1 and 2)** presented with symptomatic, persistent AF **(Figure 3)**, with resulting cardiomyopathy related to his tachyarrhythmia. His associated comorbidities included diabetes, deep vein thrombosis, obesity, and severe venous insufficiency with chest and abdominal wall varicosities. His exertional dyspnea and dilated left ventricular dysfunction (ejection fraction: 20%) prompted referral for possible combined surgical and electrophysiological treatment.

Following careful review in our multidisciplinary AF center, we elected to perform an open full Cox maze IV procedure. The ascending aorta and superior vena cava (SVC) were cannulated within purse-string sutures and umbilical tape placed around the SVC. Next, cardiopulmonary bypass was initiated. The right pulmonary veins were ablated with seven applications using the Isolator Synergy bipolar radiofrequency (RF) clamp (Atricure, Mason, OH, USA). After initiating cardiopulmonary bypass, with intermittent SVC occlusion while the heart continued beating, we ablated the left pulmonary veins six times and confirmed entrance block.

Under cardioplegic arrest, we opened the left atrium and connected the pulmonary veins with roof and floor lesions between the respective superior and inferior veins using the cryoICE^®^ cryoenergy instrument (Atricure, Mason, OH, USA). We then initiated a cryo lesion into the left atrial appendage and to the P2 leaflet of the mitral valve annulus. We further created an epicardial cryo lesion at the mitral valve annulus and a right transatrial lesion across the floor of the interatrial septum, respectively.

We excluded the left atrial appendage with a 45-mm Atricure clip (Atricure, Mason, OH, USA). Two RF ablations were then created across the middle of the posterior left atrium to further segment this large region of tissue. After completing the left-sided lesion, we closed the left atrium and released the cross-clamp. While the heart was beating, we created cryolesions to the tricuspid valve at both the anterior and posterior leaflets. A connecting lesion was made across the floor of the right atrium into the coronary sinus to connect to the transseptal lesion. We then used bipolar energy to create a lesion from the SVC to the IVC.

After closing the right atrium, a Webster^®^ deflectable decapolar catheter (Biosense Webster, Diamond Barr, CA, USA) was placed into the coronary sinus through a separate purse-string suture and verified by fluoroscopy. Next, a second purse-string suture was placed in the right atrium and a percutaneous needle was positioned through the right atrial free wall, across the intra-atrial septum and into the left atrium using transesophageal echocardiography guidance. Using the Seldinger technique, a 9-French, 16-cm Radifocus^®^ Introducer sheath (Terumo, Somerset, NJ, USA) was then positioned across the septum and secured with a purse-string suture to the right atrial free wall.

**Figure 4:**
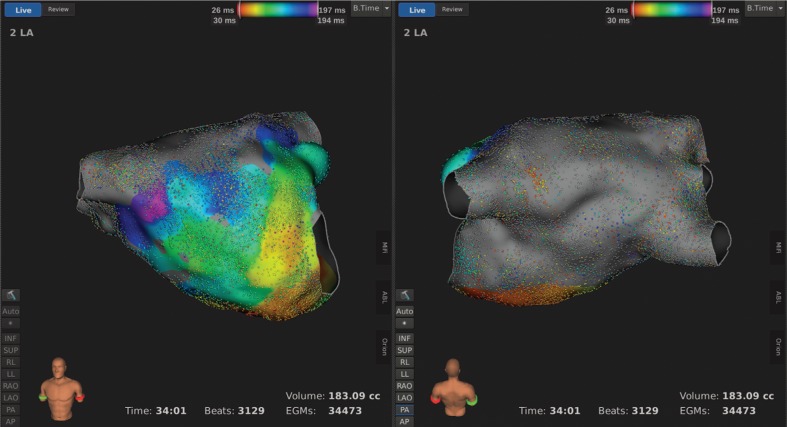
Left atrial endocardial activation map during coronary sinus pacing after the Cox maze IV procedure illustrating complete isolation of the left atrial posterior wall and pulmonary vein pedicle with voltage readings of less than 0.01 mV (gray region). From left to right: anteroposterior and posteroanterior views.

**Figure 5:**
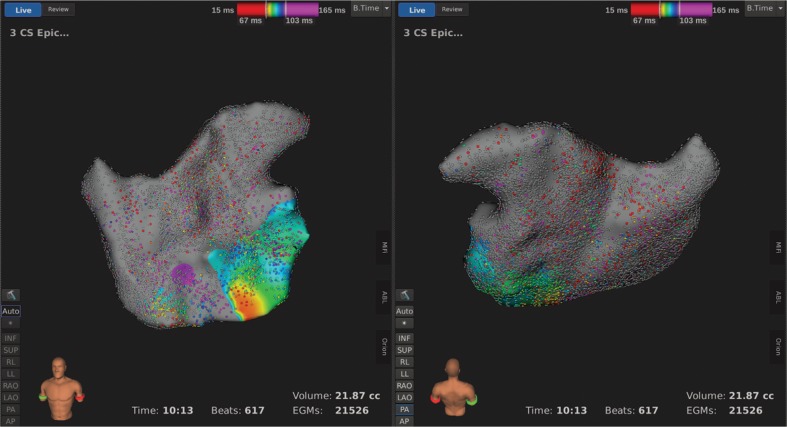
Activation map during coronary sinus pacing after the Cox maze IV procedure illustrating the epicardial left atrium with minimal activity (gray depicts less than 0.01 mV). From left to right: anteroposterior and posteroanterior views.

**Figure 6:**
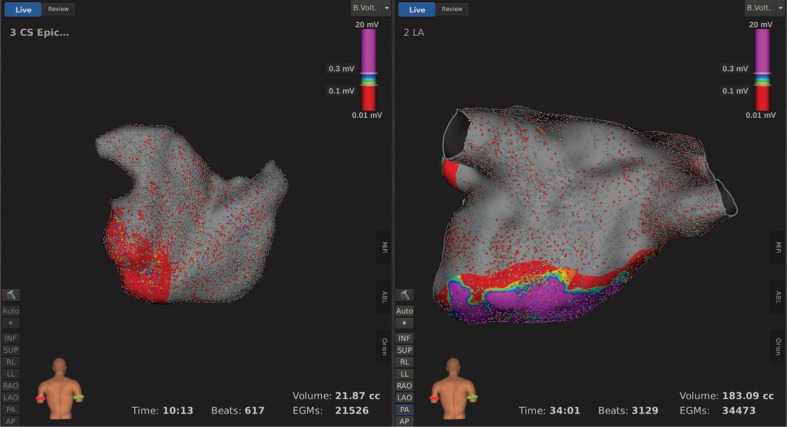
Left atrial bipolar voltage map after the Cox maze IV procedure demonstrating no significant activity in the left atrial posterior wall (0.01–20 mV; gray depicts less than 0.01 mV). From left to right: epicardial posteroanterior and endocardial posteroanterior views.

An endocardial three-dimensional (3D) map of the left atrium was constructed using the Rhythmia HDx™ hardware and software (Boston Scientific, Natick, MA, USA) and with fusion of computed tomography (CT) images **(Figure 4)**. We created an epicardial electroanatomical map using the same mapping system by manipulating the catheter around the epicardial surface **(Figure 5)**. These maps revealed complete isolation of the posterior wall of the left atrium and the pulmonary vein pedicle. There was complete block across the mitral isthmus. This demonstration of the endocardial and epicardial completeness of the Cox maze IV procedure indicated no necessity to add further RF lesions to complete the operation **(Figure 6)**.

The patient had an uneventful postoperative course and remained in normal sinus rhythm with first-degree atrioventricular block throughout his hospital stay. He continued on home-dose amiodarone, and coumadin was restarted prior to discharge. At the six-month follow-up, the patient remained on amiodarone and coumadin while demonstrating resolution of his AF. His follow-up transesophageal echocardiogram demonstrated improved left ventricular function (ejection fraction: 48%), with no significant change in his left atrial size (left atrial ejection fraction was not documented).

## Discussion

AF profoundly increases the morbidity, mortality, and health-related expenditures of those affected. Morbidities include increased risks of adverse cardiovascular outcomes such as heart failure and stroke and deleterious effects on quality of life, functional status, and cognition.^5^

The Cox maze procedure has been proven to be the most effective surgical procedure available to treat AF to date.^6^ It is a highly complex operation that divides the atria by multiple electrically isolating incisions. Further, it also isolates the pulmonary veins, which have been identified as the main AF-inciting regions.^7^ Overall, biatrial ablation surgical procedures are more effective in controlling AF as compared with procedures limited to the left atrium.^8^

Traditionally, the Cox maze III procedure employs a “cut-and-sew” technique. However, with the introduction of novel energy technologies, the Cox maze IV procedure incorporates energy sources such as RF and cryotherapy instead of a complex series of cardiac incisions. These energy sources induce scar formation and shorten procedure time. However, the maintenance of sinus rhythm following non–“cut-and-sew” Cox maze procedures has been less notable than the success rate of the traditional “cut-and-sew” approach.^7^ One theory underlying the discrepancy in outcomes is that it is difficult to achieve electrically complete linear lesions with non–“cut-and-sew” energy sources, thereby resulting in the formation of scar that is not transmural and, therefore, does not achieve complete electrical isolation. Although intraoperative scar formation can be visually assessed, electrophysiological techniques confirming the absence of electrical activity at the linear lesions are not currently routinely performed at the time of surgery.^7^

Accurate navigation inside the left atrium is required for the successful electroanatomical mapping and ablation of AF. This can be obtained using standard fluoroscopy or, more commonly, with mapping systems that combine anatomic and electrical information by point-by-point catheter electrogram acquisition. This allows an accurate anatomical reconstruction of a 3D shell of the targeted cardiac chamber.^9^ Sohns et al. in a report on 56 acquired maps, concluded that the Rhythmia HDx™ software (Boston Scientific, Natick, MA, USA) showed highly detailed endocardial electrical activation and voltage maps without the need for manual reannotation to reach procedural endpoints. Furthermore, the authors determined that the 3D visualization of the mini-basket catheter in electroanatomical mapping corresponded well to fluoroscopic findings.^10^ A different series by Ballesteros et al. drew similar conclusions, as they were able to create a map of the left atrium and pulmonary vein with a very high point-density using the Rhythmia HDx™ software (Boston Scientific, Natick, MA, USA) that was comparable with maps obtained by CT. Their study ultimately validated the anatomical precision of the Rhythmia HDx™ mapping system (Boston Scientific, Natick, MA, USA) through the quantification of heart chamber volume calculations.^11^

We present a case in which we performed a Cox maze IV procedure with simultaneous application of the Rhythmia HDx™ electroanatomical mapping system (Boston Scientific, Natick, MA, USA) at the time of operation. Through this novel approach, we were able to verify the completeness of the lesions created and reach a procedural endpoint confirmed by epicardial and endocardial maps demonstrating electrical isolation of the posterior wall of the left atrium and the pulmonary vein pedicle **(Figures 4–6)**.

In our case, we were not able to achieve percutaneous intracardiac access due the absence of an IVC in this particular patient. However, by performing the surgical ablation through sternotomy and using mapping technology, we overcame this anatomic challenge. Congenital anomalies involving the IVC have been reported in 0.3% of the general population and in 0.6% to 2% of patients with other cardiovascular defects. Congenital variations of IVC, occurring during abnormal embryological development, are usually clinically asymptomatic and detected as radiologic incidental findings. A preoperative diagnosis of interrupted IVC can help in planning for surgeries involving cardiopulmonary bypass and/or the IVC, abdominal aorta, and renal transplantation as previously described in the literature. Such also helps in the management of associated diseases like deep vein thrombosis.^12^

This case demonstrates the potential of a collaborative relationship between surgeon and electrophysiologist to best address this patient’s unique presentation. In terms of the simultaneous use of electroanatomical mapping technologies at the time of the Cox maze procedure, studies with long-term follow-up data are needed to better assess the implications of the acute confirmation of surgical ablation on outcomes and benefits. Acute confirmation of pulmonary vein isolation does not portray life-long lesion integrity.^13,14^ In fact, Accord et al.^15^ reported an autopsy series of three patients who, like our patient, had confirmed pulmonary vein exit block concomitantly with cardiac surgery and cardioplegic arrest. Despite the clinical success of patients in their series (67%–76% were in sinus rhythm at 15.2 months), only 23% of the autopsy samples demonstrated transmural lesions. Substrate modification represents one argument for clinical success in excess of anatomical lesion completeness.^13,16^ However, one must pursue contiguous and continuous transmural lesions. Failure to isolate the veins and the posterior left atrium during the initial procedure virtually guarantees nontransmural gaps in the future and predisposes the patient to supplemental tachyarrhythmias. Additional studies should focus on the maintenance of sinus rhythm over long-term follow-up without the use of antiarrhythmic medications in relation to acute lesion integrity.
